# Supplementary figures and images for: Radiation-driven acceleration in the expanding WR140 dust shell
Source: Nature. 2022 Oct 12;610(7931):269–72. doi: 10.1038/s41586-022-05155-5 (PMC9556302; doi:10.1038/s41586-022-05155-5)

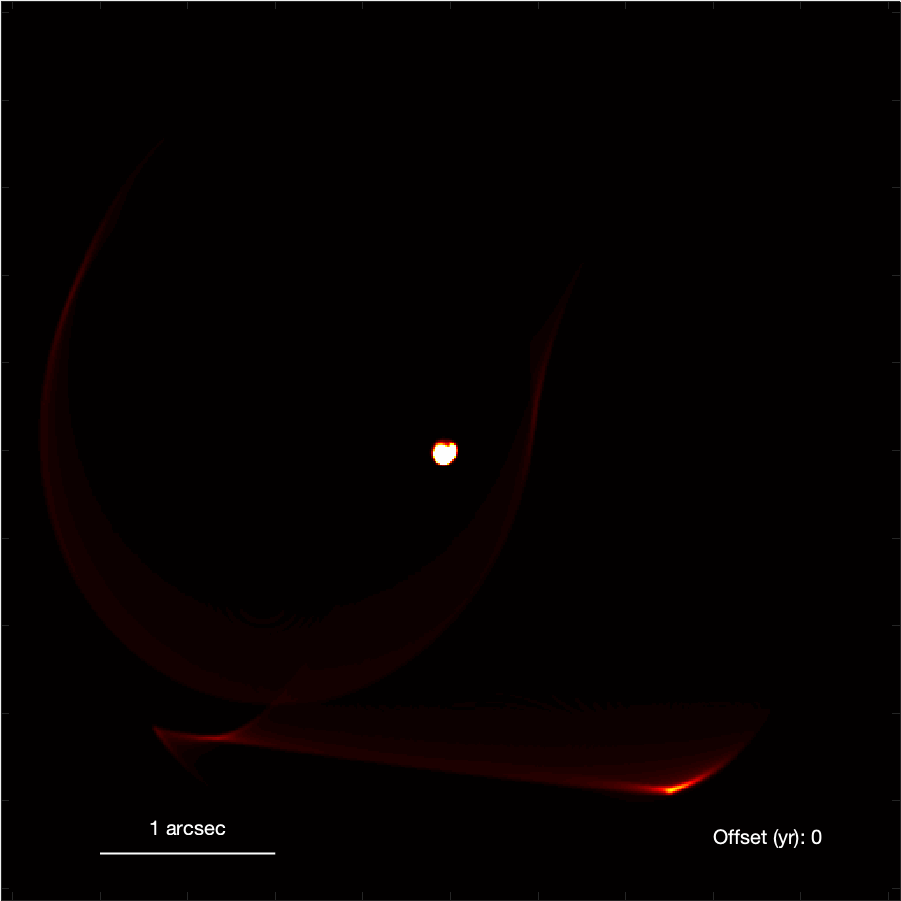

Supplement: Supplementary file 2 — This animation shows the geometric model of WR140 evolving in time. The offset value refers to the time since periastron passage. The expansion speed of the dust plume is fixed at 2,400 km s−1. The model takes into account variations in the dust production rate along the orbital and azimuthal directions. [file 41586_2022_5155_MOESM2_ESM.gif]
